# Heterodimerization of two pore domain K^+^ channel TASK1 and TALK2 in living heterologous expression systems

**DOI:** 10.1371/journal.pone.0186252

**Published:** 2017-10-10

**Authors:** Yoshiaki Suzuki, Kanako Tsutsumi, Tatsuya Miyamoto, Hisao Yamamura, Yuji Imaizumi

**Affiliations:** Department of Molecular & Cellular Pharmacology, Graduate School of Pharmaceutical Sciences, Nagoya City University, Nagoya, Japan; Xuzhou Medical College, CHINA

## Abstract

Two-pore-domain K^+^ (K_2P_) channels sense a wide variety of stimuli such as mechanical stress, inhalational anesthetics, and changes in extracellular pH or temperature. The K_2P_ channel activity forms a background K^+^ current and, thereby, contributes to resting membrane potentials. Six subfamilies including fifteen subtypes of K_2P_ channels have been identified. Each K_2P_ channel molecule with two pores consists of a homodimer of each subtype. In addition, a few heterodimers mainly within the same subfamilies have been found recently. In the present study, the possibility of heterodimerization between TASK1 (TWIK-Related Acid-Sensitive K^+^ channel) and TALK2 (TWIK-Related Alkaline pH-Activated K^+^ channel) was examined. These channels belong to separate subfamilies and show extremely different channel properties. Surprisingly, single molecular imaging analyses in this study using a total internal reflection microscope suggested the heterodimerization of TASK1 and TALK2 in a pancreatic cell line, QGP-1. This heterodimer was also detected using a bimolecular fluorescence complementation assay in a HEK293 heterologous expression system. Fluorescence resonance energy transfer analyses showed that the affinity between TASK1 and TALK2 appeared to be close to those of homodimers. Whole-cell patch-clamp recordings revealed that TASK1 currents in HEK293 cells were significantly attenuated by co-expression of a dominant-negative form of TALK2 in comparison with that of wild-type TALK2. The sensitivities of TASK1-TALK2 tandem constructs to extracellular pH and halothane were characterized as a unique hybrid of TASK1 and TALK2. These results suggested that heterodimerization of TASK1 and TALK2 provides cells with the ability to make multiple responses to a variety of physiological and pharmacological stimuli.

## Introduction

Some types of K^+^ channels play obligatory roles in the formation of resting membrane potentials by pulling it to the direction of the K^+^ equilibrium potential. It has been reported extensively that two-pore-domain K^+^ (K_2P_) channels provide background currents in various tissues, such as neurons [1], heart [2], vascular smooth muscle [3], and pancreas [4, 5]. K_2P_ channels consist of fifteen subtypes classified into six subfamilies. Each subtype of K_2P_ channel contains the two pore domains and forms a homodimer [6]. Activation of K_2P_ channels occurs in response to a number of stimuli, such as mechanical stress, inhalational anesthetics, and changes in extracellular pH or temperature, and modulates membrane potentials to induce physiological responses [6].

One of the key mechanisms of K_2P_ dimerization is the formation of a disulfide bond between Cys localized the Cap region connecting the M1 and pore regions [7]. This Cys is conserved in K_2P_ channels except for the TASK (TWIK-related acid-sensitive K^+^ channel, i.e. TASK1, TASK3, and TASK5) and THIK (tandem pore-domain halothane inhibited K^+^ channel, i.e. THIK1 and THIK2) subfamilies [8]. It has been reported that hydrophobic interactions between coiled-coil domains in the Cap domain are important for dimerization between TASK channels [8].

Moreover, some K_2P_ channel members form heterodimers as well as homodimers. In most cases, however, heterodimerization happens within the same subfamilies, such as TASK1-TASK3 [9], TREK1-TRAAK [10], and THIK1-THIK2 [11]. In fact, heterodimerization between different subfamilies has not been reported, except for TWIK1 [12, 13]. Heterodimerization appears to have a major physiological impact in expanding the functional diversity of K_2P_ channels.

In the present study, we focused on K_2P_ channels that belong to distinct subfamilies, TASK1 and TALK2 (TWIK-related alkaline pH-activated K^+^ channel 2). These channels showed similar tissue expression, such as that in the pancreas, heart, brain, lung, and placenta [1, 14]. Both of these proteins are sensitive to extracellular pH and suppressed by low pH, whereas their sensitivity is distinct; the IC_50_ of TASK1 is pH 7.3–7.6 and that of TALK2 is pH 8.8 [15]. TASK1 regulates action potential formation in neurons [16] and the heart [2], cell proliferation and apoptosis in cancer cells [17], and hormonal secretion in the pancreas [4, 5]. On the other hand, the physiological significance of TALK2 has not yet been established. A gain-of-function mutation in TALK2 (Gly88Arg) was identified as a novel arrhythmia gene [18]. It is also unclear whether TALK2 forms heterodimers with other subtypes of K_2P_.

Using imaging and electrophysiological analyses, we revealed heterodimerization of TASK1 and TALK2 in HEK293 cells and a pancreatic islet cell line, QGP-1. This heterodimer showed different electrophysiological properties from the two homodimer forms. Thus, heterodimerization among different subfamilies may increase the functional variation of K_2P_ channels.

## Materials and methods

### Cell culture

HEK (human embryonic kidney) 293 cell and QGP-1 (a human cell line derived from pancreatic islet cell carcinoma) were supplied from the Japanese Collection of Research Bioresources (JCRB) Cell Bank (Osaka, Japan). HEK293 and QGP-1 cells were maintained at 37°C, in 5% CO_2_ with high glucose DMEM (HEK293) or RPMI-1640 (QGP-1) (Wako, Osaka, Japan) containing 10% fetal bovine serum (Invitrogen, Carlsbad, CA), 100 units/ml penicillin (Wako), and 0.1 mg/ml streptomycin (Meiji Seika, Tokyo, Japan).

### Electrophysiological recordings

Electrophysiological studies were performed as described previously [19]. For measurements of whole-cell currents, the pipette solution contained: (mmol/l) 140 KCl, 4 MgCl_2_, 10 HEPES, 0.05 EGTA, and 2 Na_2_ATP. The pH was adjusted to 7.2 with KOH. The extracellular solution had an ionic composition (mmol/l) of: 137 NaCl, 5.9 KCl, 2.2 CaCl_2_, 1.2 MgCl_2_, 14 glucose, 10 TEACl, 10 MES (pH 5.4 and 6.4) or 10 HEPES (pH 7.0 and 7.4) or 10 EPPS (pH 8.0 and 8.4) or 10 CHES (pH 9.4, 9.7 and 10.0). The pH was adjusted with NaOH. Whole-cell currents were activated from a holding potential of -80 mV by applying 300-ms ramp pulses, once every 5 s, to a voltage range between -100 and +100 mV. Data were sampled at 5 kHz and filtered at 1 kHz.

### Plasmid constructs and transfection

The full-length of cDNAs encoding the human KCNK3 (TASK1: NM_002246.2), KCNK17 (TALK2: NM_031460.3) and KCNK15 (TASK5: NM_022358.3) were labeled with fluorescent proteins (CFP, GFP, YFP, or mCherry, Clontech Laboratories, Mountain View, CA) at the intracellular N- or C-termini. In the case of bimolecular fluorescence complementation (BiFC) analysis, the N- or C-termini of K_2P_ channel was labeled by fragments of the N- (1–173: VN173) or C- (155–238: VC155) termini of Venus (pBiFC-VN173 and pBiFC-VC155, Addgene plasmid #22010 and #22011, respectively) [20]. A dominant negative form of TALK2 (TALK2(DN)) was produced by substitution of Gly with Glu (G118E) in the selectivity filter (^118^GYG^120^) of the first pore domain. A TASK1-TALK2 tandem construct was created by binding the C-terminus of TASK1 and N-terminus of TALK2 via a GGGGSGGGGSGGGGS linker. GFP was fused to the C-terminus of TALK2. A TASK1-TALK2(DN) tandem construct was also produced by introducing the same point mutation as TALK2(DN) into the TASK1-TALK2 construct. HEK293 cells were transiently transfected with cDNA (total ~2 μg) using polyethylenimine (PEI, Polysciences, Warrington, PA) or Lipofectamine 2000 (Invitrogen). Experiments were preformed 24–72 h after transfection. All constructs were confirmed by DNA sequencing.

### Single-molecular imaging

Single-molecular imaging was performed using a total internal reflection fluorescence (TIRF) imaging system (Nikon, Tokyo, Japan), as described previously [19, 21]. GFP- or mCherry-fused target proteins were excited with 488-nm or 561-nm lasers (Nikon). GFP/mCherry emission data were collected using a combination of dichroic mirrors and dual band-pass filters (505–530/570–660 nm; Omega Optical, Brattleboro, VT). The resolution of images was 105 nm per pixel (x–y) and less than 200 nm (z). All experiments were carried out at room temperature (25°C).

### FRET analysis

The efficiency of FRET (E_FRET_) was evaluated based on the acceptor photobleaching method, in which the emission of the donor fluorophore is compared before and after photobleaching of the acceptor [19]. Fluorescence was observed using a laser scanning confocal fluorescent microscope (A1R, Nikon) equipped with a fluorescent microscope (ECLIPSE Ti, Nikon), an objective lens (Plan Apo 60× 1.40 NA, Nikon), and NIS Elements software (version 3.10, Nikon). The excitation wavelength from the multi-argon laser for YFP was 514 nm and the emission light was collected by a band-pass filter (DM514/BA525–555). CFP was excited at 457 nm and fluorescence was measured using a filter cube (DM457/BA465–500). Images were acquired at 1024×1024 pixel (0.14 μm/pixel). Regions of interest (ROIs) were set on the cells exhibiting both YFP and CFP fluorescence. The fluorescence of YFP was photobleached using a multi-argon laser (514 nm) for 1.5 min. E_FRET_ was calculated as the percentage increase in CFP emission after YFP photobleaching, i.e. E_FRET_ (%) = [(CFP_after_-CFP_before_)/CFP_after_] × 100, where CFP_after_ and CFP_before_ are CFP fluorescence in ROIs after and before YFP photobleaching, respectively. Bleaching efficiency was also calculated using a following equation; E_bleach_ (%) = [(YFP_before_-YFP_after_)/YFP_before_] × 100, where YFP_before_ and YFP_after_ are YFP fluorescence before and after photobleaching, respectively. Data were discarded when E_bleach_ (%) was less than 40%.

### Immunostaining

Cells were immunostained as described previously [21]. QGP-1 cells were labeled with anti-TASK1 antibody (1:100 dilution, APC-024, Alomone Labs, Jerusalem, Israel) or anti-TALK2 antibody (1:50 dilution, sc-390435, Santa Cruz Biotechnology, Dallas, TX) for 12 hours at 4°C after fixation and permeabilization. Then, cells were washed and incubated with Alexa488-conjugated anti-rabbit IgG (for anti-TASK1) or Alexa488-conjugated anti-mouse IgG (for anti-TALK2) at 1:500 dilution for 1 hr. After washing, fluorescently labeled cells were observed using a confocal microscope system mentioned above. The excitation wavelength from the multi-argon laser for Alexa488 was 488 nm and the emission light was collected by a band-pass filter (DM488/BA500–530).

### RNA extraction and reverse transcription PCR

Total RNA extraction from QGP-1 cells was performed as reported previously [19]. Human total RNA was purchased from BioChain (Newark, CA). Using the extracted total RNA, reverse transcription was performed using ReverTra ACE qPCR RT Master Mix (TOYOBO, Osaka, Japan). Primer pairs flanked an intron to avoid contamination from genomic DNA. The sequences of the primers were as follows; TASK1 (NM_ NM_002246, 1004–1123): 5'-ACT ACG GAC ACC GCC TCA TC-3' and 5'-CTT CTC GCG GCT CTT GTA CC-3', TALK2 (NM_031460.3, 311–430): 5'-CGA CAA GTG GGA GCT GTT G-3' and 5'-CTG GTG GTG TTG CTG AGG AG-3', GAPDH (NM_001289746.1, 670–802): 5’-GAC AAC TTT GGT ATC GTG GAA GG-3’ and 5’-AGG CAG GGA TGA TGT TCT GG-3’. The reverse transcription-polymerase chain reaction (RT-PCR) amplification was carried out using KOD-Plus-Neo (TOYOBO, Tokyo, Japan) and a GeneAmp PCR System 2700 (Applied Biosystems). Gene products were analyzed by 2.0% agarose gel electrophoresis.

### Western blotting

Cells were lysed in RIPA Buffer (Sigma-Aldrich) with protease inhibitor cocktail (Sigma-Aldrich). Homogenates were centrifuged (15000 × g, 30 min, 4°C) and the protein concentration of the supernatant was calculated using a DC protein assay (Bio-Rad, Hercules, CA). The lysates were boiled with 5×sample buffer with 2-mercaptoehanol and finally subjected to SDS-PAGE (7.5%). The blots were blocked using blocking buffer (Beacle, Kyoto, Japan) for 12 hr at 4°C, and then incubated with anti-TASK1 or anti-TALK2 antibodies for 12 hr at 4°C. After washing, the blots were incubated with anti-rabbit or anti-mouse horseradish peroxidase-conjugated IgG (Chemicon International, Temecula, CA). An enhanced chemiluminescence detection system (GE Healthcare Biosciences, Piscataway, NJ) was used to obtain images of the bound antibody. The resulting images were analyzed using a LAS-3000 device (Fujifilm, Tokyo, Japan).

### Co-immunoprecipitation (Co-IP)

Co-IP procedures were carried out using a Pierce Co-Immunoprecipitation Kit according to the experimental manual supplied by Thermo Scientific [19]. Briefly, HEK293 cells were lysed in IP Lysis/Wash Buffer (0.025 M Tris, 0.15 M NaCl, 0.001 M EDTA, 1% NP-40, 5% glycerol; pH 7.4) with protease inhibitor cocktail (Sigma-Aldrich). Homogenates were centrifuged (15000 × g, 30 min, 4°C) and supernatant was precleared with control resin (1 h, 4°C). Precleared lysates (~ 0.5 mg of protein) were incubated with AminoLink Plus Coupling Resin, with which 10 μg anti-GFP antibody (mFX73, Wako) was immobilized (overnight, 4°C). The incubated lysates were washed with IP Lysis/Wash Buffer, eluted with Elution Buffer, boiled with 5× sample buffer with 2-mercaptoehanol and finally subjected to SDS-PAGE (10%). The blots were incubated with anti-TASK1 antibody and then incubated with anti-rabbit horseradish peroxidase-conjugated IgG (Chemicon International, Temecula, CA). An enhanced chemiluminescence detection system (GE Healthcare Biosciences, Piscataway, NJ) was used for getting images of the bound antibody. Resulting images were analyzed by a LAS-3000 device (Fujifilm, Tokyo, Japan).

### Chemicals and solutions

1-bromo-1-chloro-2,2,2-trifluoroethane (halothane) was purchased from Alfa Aesar (Heysham, UK).

### Statistics

Pooled data are shown as the mean ± s.e.m. Statistical significance between two groups was evaluated using Student’s t-test after F-test application. Tukey’s test or Dunnett’s test following one way ANOVA was examined for statistical analyses among multiple groups.

The effect of pH on currents was examined by plotting currents measured at membrane potential of +100 mV against extracellular [H^+^]. Fitting of a Hill equation to the data was done for each individual experiment. The parameters were defined in the following equation: y = 1/[1+([H^+^]/[K_1/2_])^n^], where K_1/2_ is the concentration giving a half-maximal response, and n is the Hill coefficient. For graphical representation, mean ± s.e.m. of normalized current values were obtained from individual experiments and plotted with curves constructed with average parameters obtained from the individual fits or with the sum of TASK1 and TALK2 curves, i.e. y = s/[1+([H^+^]/[K_1/2(TASK1)_])^n(TASK1)^]+ (1-s)/[1+([H^+^]/[K_1/2(TALK2)_])^n(TALK2)^], where s is the ratio of TASK1 current component to the total current. Fits were done by using the Origin6.0 software (LightStone, Tokyo, Japan).

## Results

### Expression profile of TASK1 and TALK2 in human tissues and a pancreatic cell line

**Fig 1:**
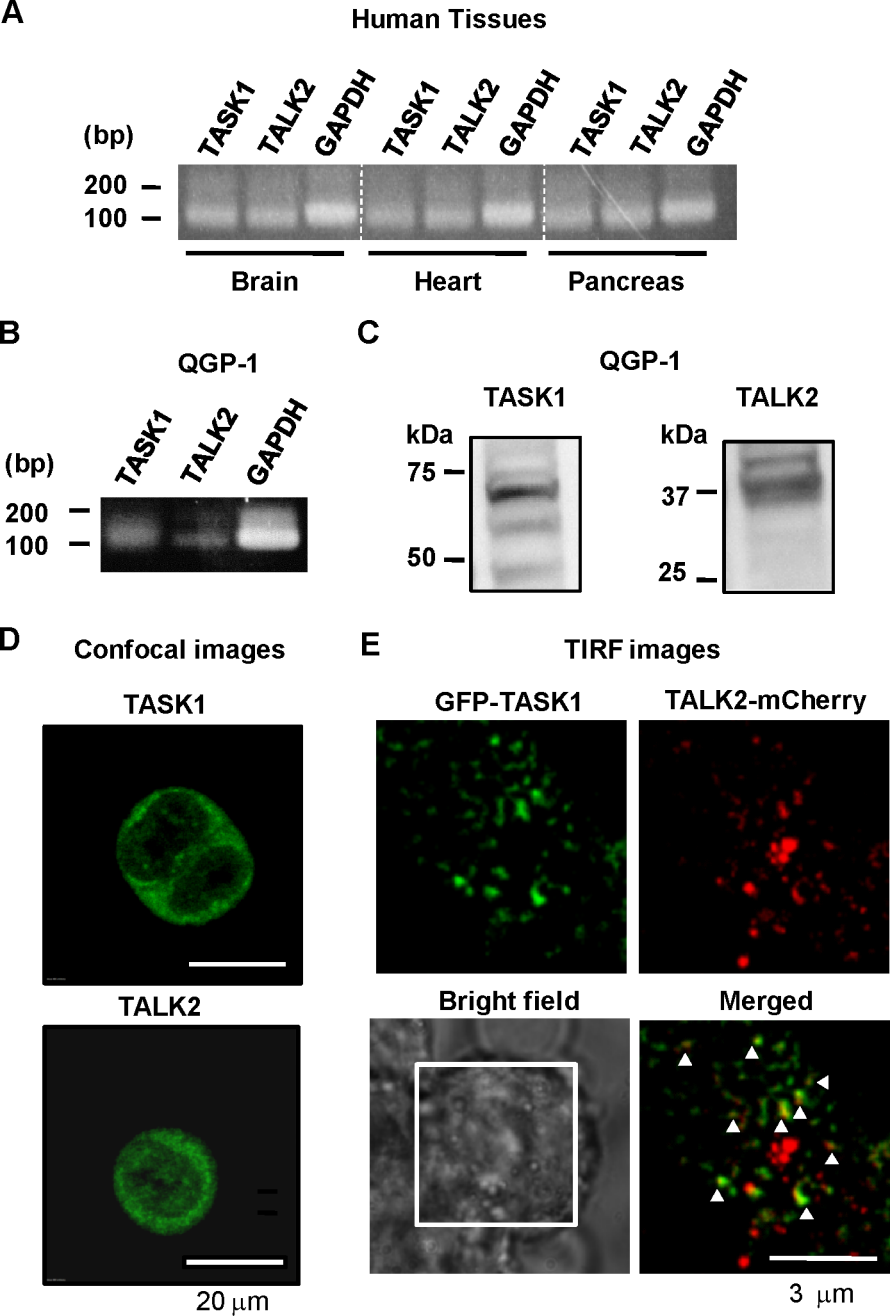
Expression profiles of TASK1 and TALK2 in human tissues and a pancreatic cell line. **(A and B)** TASK1 and TALK2 mRNA is expressed in (i) human brain, heart, and pancreas, and (ii) a human pancreatic cell line, QGP-1. The sizes of bands corresponding to TASK1 and TALK2 are 119 bp. **(C)** Western blotting was performed to confirm TASK1 and TALK2 protein expression in QGP-1 cells. Proteins from whole cell lysates (20 μg/lane) were subjected to SDS-PAGE. Each protocol was repeated three times and showed very similar results. **(D)** Immunostaining images of TASK1 (upper) and TALK2 (lower) in QGP-1 cells. Scale bar corresponds to 20 μm. Representative images from three independent experiments were shown. **(E)** TIRF images from QGP-1 cells expressing GFP-TASK1 and TALK2-mCherry (cells were transfected with cDNA at a ratio of 1:1). Signals from GFP, mCherry and co-localization of these signals (indicated by triangles) are illustrated by green, red, and yellow, respectively. Representative images from 8 cells are shown.

First, mRNA expression of TASK1 and TALK2 in human tissues and pancreatic cell line QGP-1 was determined by RT-PCR. As shown Fig 1, the bands corresponding to TASK1 or TALK2 were clearly detected in cDNA from human brain, heart, and pancreas (Fig 1A), as well QGP-1 cells (Fig 1B). Then, the protein expression of these channels in QGP-1 cells was examined by western blotting (Fig 1C) and immunostaining assays (Fig 1D). Specific antibodies against each channel revealed the protein expression of TASK1 and TALK2 in QGP-1. To visualize molecular coupling between TASK1 and TALK2, GFP-TASK1 and TALK2-mCherry were transiently expressed in QGP-1 cells and detected using single molecular imaging analysis with a TIRF microscope (Fig 1E). Co-localization of these channels (yellow particles in Fig 1E) was clearly detected. These results suggested that TASK1 and TALK2 are expressed and thought to make heterodimers in human tissues.

### Fluorescence imaging revealed heterodimerization of TASK1 and TALK2 in a HEK293 heterologous system

**Fig 2:**
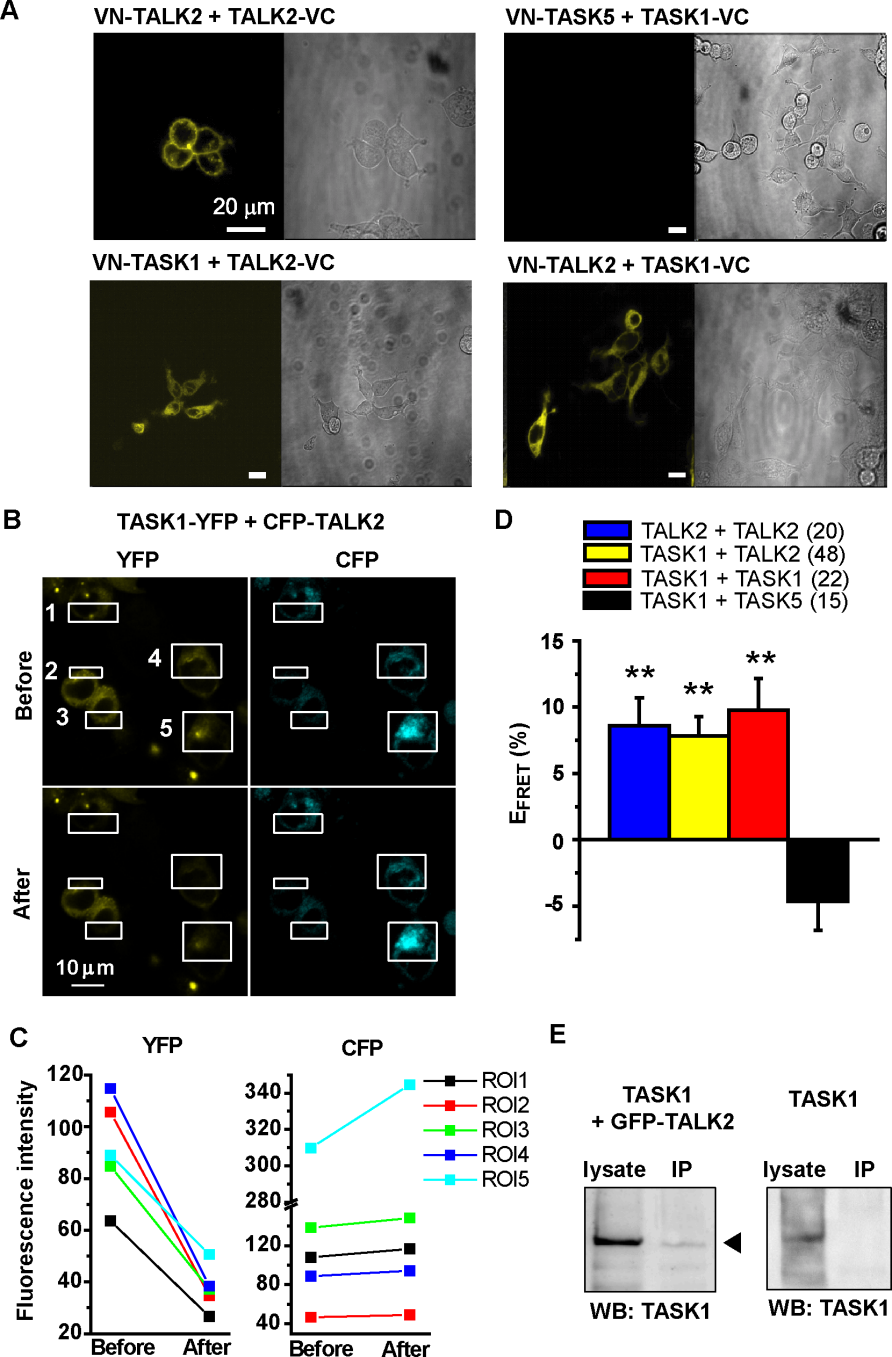
Image analyses of heterodimerization between TASK1 and TALK2. **(A)** A BiFC assay was performed to detect direct coupling between TASK1 and TALK2. A representative set of images from three independent experiments are presented. HEK293 cells expressing VN-TALK2+TALK2-VC or VN-TASK5+TASK1-VC were used as positive and negative controls, respectively. **(B)** FRET analysis based on acceptor photobleaching was carried out using a confocal microscope. YFP within the ROI 1–5 (white rectangles in the figures) in HEK293 cells co-expressing TASK1-YFP+CFP-TALK2 was selectively photobleached using a laser at 514 nm wavelength. **(C)** Fluorescence intensity of YFP and CFP in the ROIs was obtained and compared before and after YFP photobleaching. Note that the increase in CFP fluorescence, i.e. the cancellation of FRET, was detected together with the decrease in YFP fluorescence. E_FRET_ values (calculated based on an equation mentioned in Materials and Methods) of ROI 1–5 are 7.4, 4.8, 6.6, 6.0 and 10.1%, respectively. **(D)** E_FRET_ values were compared between cells co-expressing TALK2-YFP+CFP-TALK2, TASK1-YFP+CFP-TASK1, TASK1-YFP+CFP-TALK2, and TASK5-YFP+CFP-TASK1. Cells were transfected with cDNA at a ratio for CFP:YFP of 1:1. Numbers of cells are shown in parentheses. **p<0.01 vs. CFP-TASK1+TASK5-YFP. **(E)** Co-IP assay was performed using HEK293 cells expressing GFP-TASLK2 + TASK1 (without any tag) or only TASK1 (for a negative control). Lysates were precipitated with anti-GFP antibody, and blotted using the anti-TASK1 antibody. Each protocol was repeated three times and showed very similar results.

Heterodimerization of TASK1 and TALK2 was examined using a BiFC assay in a HEK293 heterologous system [19, 20]. As shown in Fig 2A, Venus fluorescence was consistently detected in HEK293 cells co-expressing TASK1 and TALK2 tagged with the N- (VN173) or C- (VC155) termini of Venus (See VN-TASK1+TALK2-VC and VN-TALK2+TASK1-VC in Fig 2A). Cells expressing VN-TALK2 and TALK2-VC were used as a positive control. Because TASK1 and TASK5 are known not to form a heterodimer [22], cells co-transfected with VN-TASK5 and TASK1-VC were used as a negative control.

To examine further the possible interaction between TASK1 and TALK2, FRET analysis based on the acceptor photobleaching method was also carried out using a confocal microscope (Fig 2B, See also Materials and Methods). When YFP within the ROIs (white rectangles in Fig 2B) was selectively photobleached using a 514 nm laser, an increase in CFP fluorescence because of a cancellation of energy transfer from CFP to YFP, i.e. FRET, was consistently observed in cells co-expressing TASK1-YFP and CFP-TALK2 (Fig 2C). The FRET efficiency (E_FRET_) of TALK2-YFP+CFP-TALK2 was 8.6±2.1% (20 cells), which was not significantly different from that of TASK1-YFP+CFP-TASK1 (9.8±2.4%, 22 cells) and TASK1-YFP+CFP-TALK2 (7.8±1.5%, 48 cells, Fig 2D). FRET was not detected in cells co-transfected with TASK1-YFP+CFP-TASK5 (-4.6±2.2%, 15 cells).

Co-immunoprecipitation (Co-IP) assay was also conducted using HEK cells expressing GFP-TALK2 and TASK1 (Fig 2E). The protein lysates were precipitated with an anti-GFP antibody and blotted with an anti-TASK1 antibody. A clear band that shows protein-protein interaction between TASK1 and TALK2 was detected. In contrast, no band was detected in samples from HEK cells transfected with only TASK1.

In summary, our analyses indicated that TASK1 and TALK2 bind to form a heterodimer with presumably similar affinity to form heterodimers.

### Dominant negative mutant of TAKL2 attenuated TASK1 currents

**Fig 3:**
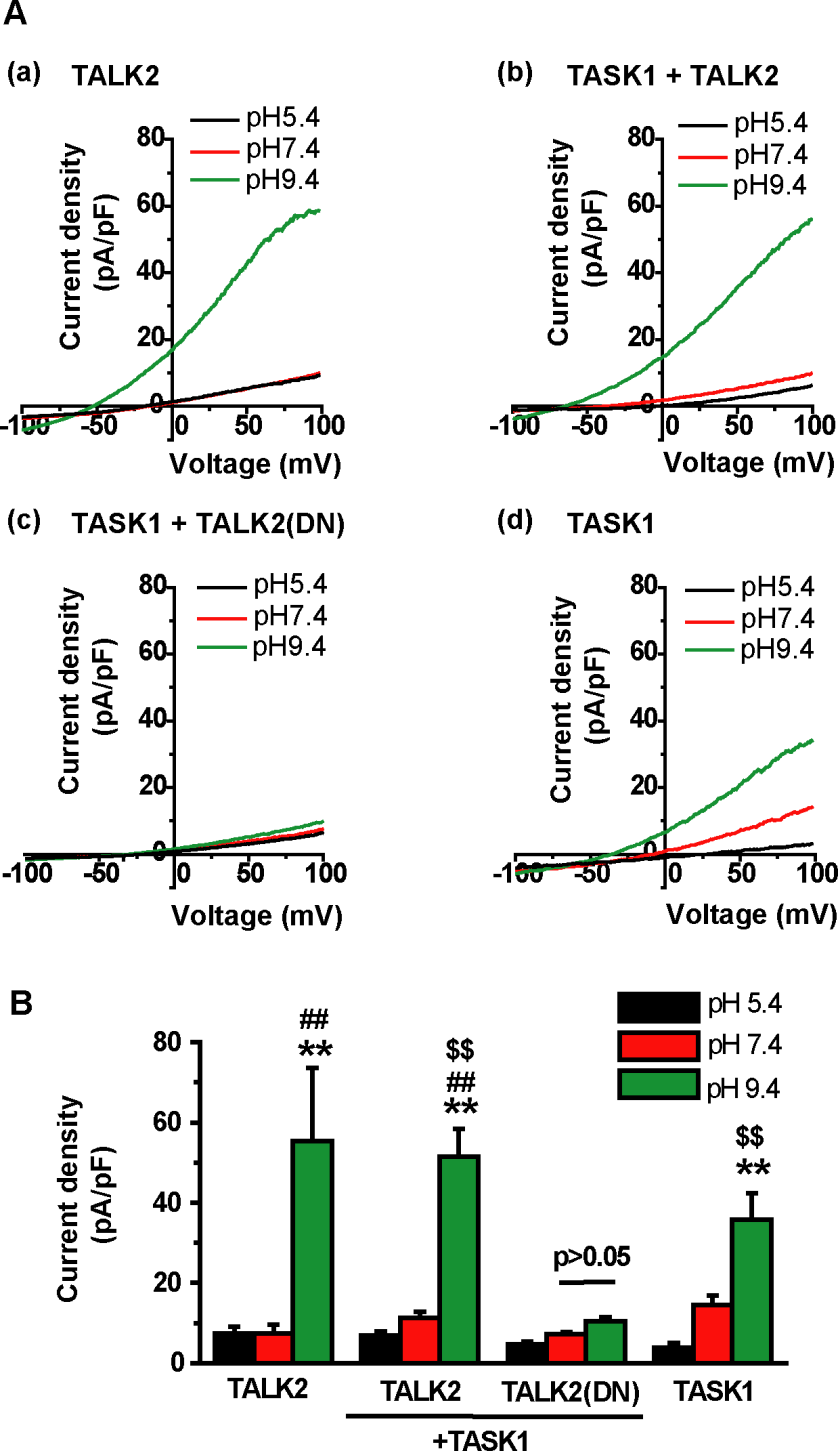
Effects of co-transfection of a dominant negative form of TALK2 on TASK1 currents. **(A)** Membrane currents were recorded using whole-cell patch-clamp techniques. Ramp pulse from +100 mV to -100 mV was applied to the cells at extracellular pH 5.4, 7.4 and 9.4. Here, constant amounts of cDNA of 0.2 μg GFP-TASK1 (A(b)-(d)), 0.6 μg TALK2-mCherry (A(a) and (b)) or 0.6 μg TALK2(DN)-mCherry (A(c)) were introduced to HEK293 cells. Therefore, cDNA ratio (TASK1:TALK2) in A(b) and A(c) was 1:3. **(B)** Averaged current density at +100 mV was compared. Number of cells in (a)-(d) was 4, 8, 8, and 6, respectively. **p<0.01 vs. pH5.4, ##p<0.01 vs. pH7.4, $$p<0.01 vs. TASK1+TALK2(DN) at pH 9.4.

Patch-clamp recordings were carried out to further examine the possibility of TASK1-TALK2 heterodimerization. Here, a dominant-negative form of TALK2 (TALK2(DN)) was introduced into HEK293 cells together with TASK1. As shown in Fig 3A, HEK293 cells expressing TASK1+TALK2(DN) did not show alkaline-activated outward currents (current density at pH 9.4, 10.4±1.0 pA/pF, 8 cells, Fig 3B). In contrast, HEK293 cells expressing TASK1+TALK2 or TASK1 alone showed significant outward currents (51.5±1.6 pA/pF, 8 cells and 35.9±6.5 pA/pF, 6 cells, respectively). These results indicated that TALK2(DN) inhibits TASK1 function by forming heterodimers.

### Tandem construct of TASK1-TALK2 showed altered electrophysiological propertie.

**Fig 4:**
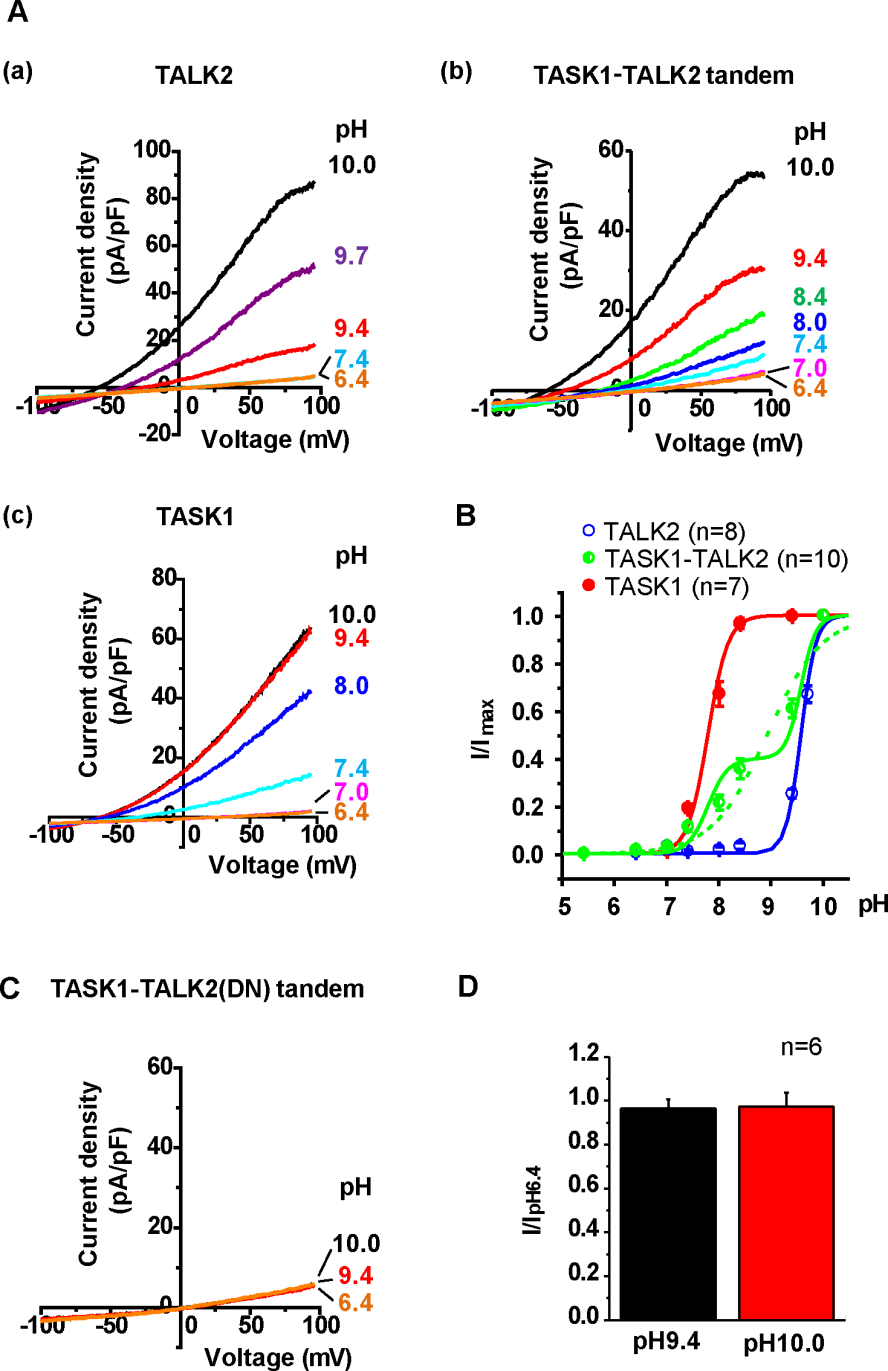
Effects of changes in extracellular pH on TASK1-TALK2 heterodimers. **(A)** pH-sensitive currents were measured using whole-cell patch-clamp recordings. HEK293 cells were transfected with GFP-TALK2 (A(a)), TASK1-TALK2-GFP (A(b)), or GFP-TASK1 (A(c)). Current traces under different pH conditions are shown. **(B)** Normalized pH-dependence curves were obtained from HEK293 cells expressing GFP-TALK2, TASK1-TALK2-GFP or GFP-TASK1. Because both TASK1 and TALK2 are completely inactive at pH of less than 6.4, the minimum current values at pH 5.4 or 6.4 (i.e. endogenous background currents of HEK293 cells) were subtracted from those at each pH value. The currents were normalized to the maximum currents. Data points were fitted by a Hill equation and were constructed by using the average of fitted parameter of the individual experiments (solid lines for TASK1 (red) and TALK2 (blue) homodimers and the dotted line for TASK1-TALK2 (green)) or by the sum of two Hill equations for the TASK1 and TALK2 curves (the green solid line for TASK1-TALK2, s = 0.4) (See Text and Materials and Methods). **(C)** pH-sensitive currents in HEK293 cells expressing TASK1-TALK2(DN) were measured at pH 6.4, 9.4 and 10.0. **(D)** The relative currents (I/I_pH6.4_ at +100 mV) at pH 9.4 and 10.0 were compared. P>0.05. vs. pH6.4 by Dunnett’s test.

To characterize the channel properties of the TASK1-TALK2 heterodimer, a tandem construct of TASK1-TALK2 was produced. Then, the pH-sensitivity of the tandem construct was examined. pK_1/2_ of extracellular acidification for TASK1 current was pH 7.3 [1], which was substantially different from that for TALK2 (pH 8.8 [23]). Each channel tagged with GFP was transiently expressed in HEK293 cells and currents were recorded at variable pH ranging from pH5.4 to 10.0 to examine pH-dependence as shown in Fig 4A and 4B. In HEK293 cells expressing TALK2, outward currents were significantly increased at pH 9.4 and higher (pK_1/2_: 9.56±0.01, Hill coefficient: 3.0±0.1 from 8 cells). On the other hand, TASK1 current was potentiated at pH 7.4 and higher (pK_1/2_: 7.7±0.1, Hill coefficient: 2.3±0.3 from 7 cells). In HEK293 cells expressing TASK1-TALK2, outward currents were evoked at higher than pH 7.4. Interestingly, the pH-dependence curve of TASK1-TALK2 showed a double sigmoid behavior rather than a simple sigmoid (a green dotted line, pK_1/2_: 8.8±0.1, Hill coefficient: 0.83±0.04 from 10 cells). The data were well fitted to the curve (green solid line) consisting of double Hill equations as follows;

y = s/[1+([H^+^]/[K_1/2(TASK1)_])^n(TASK1)^]+(1-s)/[1+([H^+^]/[K_1/2(TALK2)_])^n(TALK2)^], where s (0.4) is the ratio of TASK1 current component to the total current, p>0.05 by χ^2^ test.

We also produced TASK1-TALK2(DN) construct, and measured pH-sensitive currents. As shown in Fig 4C and 4D alkalinization of bath solutions to pH 9.4 and 10.0 did not activate K^+^ currents (I/I_pH6.4_: 0.96±0.04 at pH9.4 and 0.97±0.06 at pH 10.0 from 6 cells, Fig 4D).

Thus, TASK1-TALK2 heterodimers showed different pH-sensitivity from two homodimers.

### Tandem construct of TASK1-TALK2 showed a distinct response to halothan

**Fig 5:**
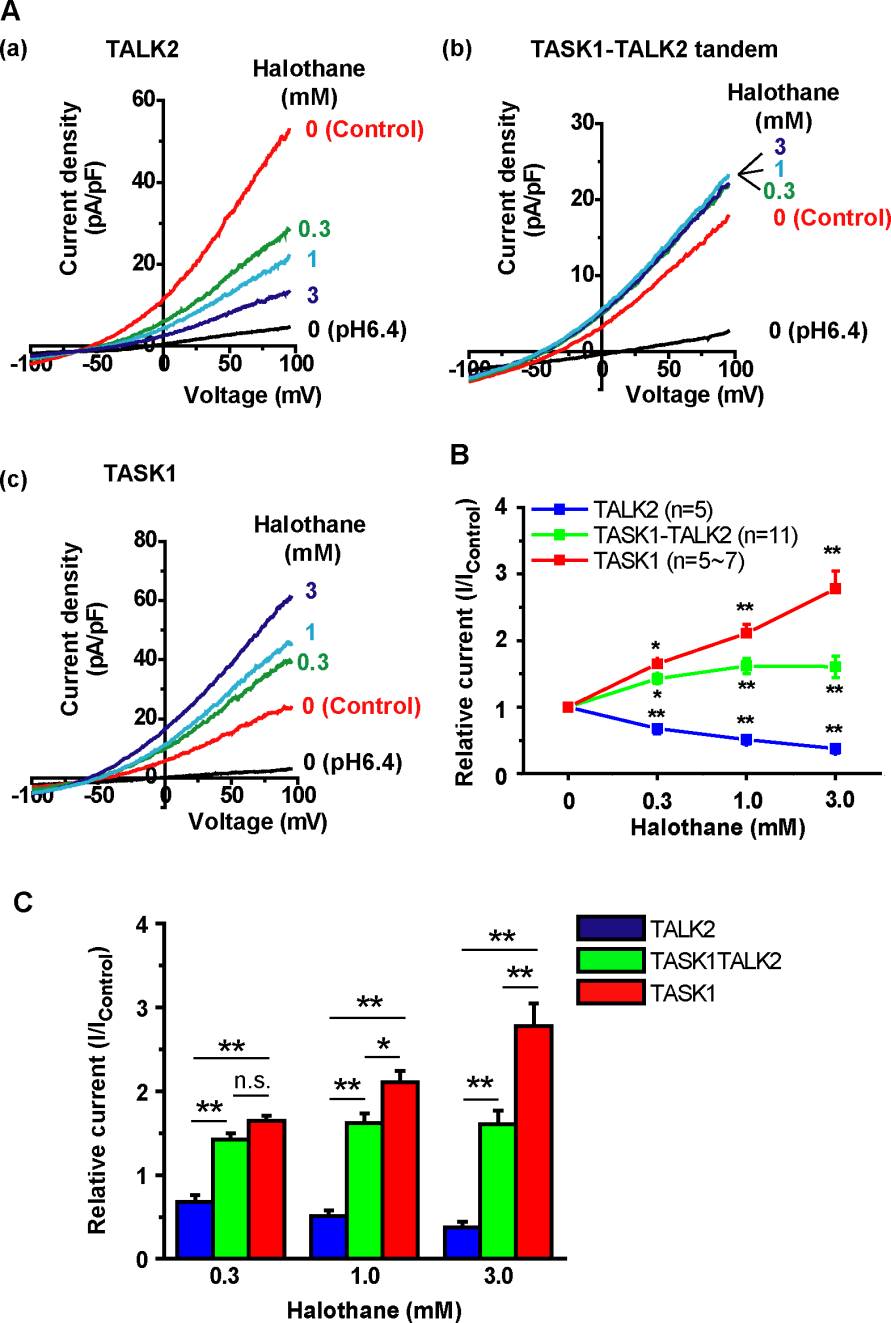
Effects of halothane on TASK1-TALK2 tandem channel. **(A)** Responses to halothane (0.3, 1 and 3 mM) were measured using whole-cell patch-clamp recordings. HEK293 cells were transfected with GFP-TALK2 (A(a)), TASK1-TALK2-GFP (A(b)), or GFP-TASK1 (A(c)). Here, the extracellular pH was set at 9.4 to elicit TALK2 currents. **(B)** The rate of increase in currents at +100 mV after treatment with each concentration of halothane. Currents at pH6.4 (i.e. background currents) were subtracted from currents at pH9.4 with or without halothane in each cell. *p<0.05, **p<0.01 by Dunnett’s test. **(C)** The effect of halothane on TALK2, TASK1-TALK2 and TASK1 was compared at each concentration. *p<0.05, **p<0.01 by Tukey’s test.

Finally, halothane sensitivity of the tandem construct was examined, because TASK1 [16, 24] and TALK2 [14] show opposing responses, i.e. halothane enhances TASK1 but it inhibits TALK2. Here, outward currents were recorded at pH 9.4, because TALK2 did not show any currents at pH 7.4. As shown in Fig 5, 0.3~3 mM halothane significantly reduced TALK2 currents (Fig 5A(a) and 5B) but, in contrast, increased TASK1 currents (Fig 5A(c) and 5B). Notably, halothane (0.3~3 mM) increased TASK1-TALK2 currents (Fig 5A(b) and 5B), but the potentiating effect on the tandem construct was significantly smaller than that on TASK1 at 1.0 and 3.0 mM (p<0.05 by Tukey’s test, Fig 5C). These results suggested that the TASK1-TALK2 heterodimer acquires distinct properties from each homodimer.

## Discussion

So far, heterodimerization between K_2P_ channels has been mainly examined within the same subfamilies such as TASK1-TASK3 [9], TREK1 or TREK2-TRAAK [10], and THIK1-THIK2 [11]. The only exception currently is TWIK1, which forms heterodimers with TASK [12] or TREK [13] subfamilies through disulfide bonds. To the best of our knowledge, information about the heterodimerization of TALK family (TALK1, TALK2 and TASK2), regardless of within the same subfamily or among different subfamilies are lacking. In the present study, heterodimerization of TASK1 and TALK2, which belong to separate K_2P_ subfamilies, was first shown not only in a HEK293 heterologous expression system but also in a pancreatic islet cell line, QGP-1.

It has been reported that TASK1 shows low plasma membrane (PM) trafficking efficiency, because the binding of p11, also known as S100A10, to the i20 domain (^295^G-^314^W) of TASK1 enhances endoplasmic reticulum (ER) retention [25]. The i20 domain also contains the binding site for the AP-2 complex (^300^YAEV^303^) and promotes endocytosis [26]. Furthermore, TASK1 has two COP-I complex binding sites (^2^KR^3^ and ^389^KRR^391^) at the N- and C-termini, respectively. Thus, TASK1 is subject to retrograde transport to the ER [27]. Conversely, the binding of 14-3-3 proteins to the C-terminus of TASK1 masks the latter domain (^389^KRR^391^), enhances ER export, and regulates intracellular trafficking [27, 28]. Although little is known about TALK2, including its intracellular dynamics, robust outward current and clear PM expression were demonstrated in Figs 2A and 3. Heterodimerization may alter the intracellular trafficking pathway compared with that of each homodimer.

Regarding dimerization between K_2P_, two mechanisms have been reported. TWIK1 forms a dimer using a disulfide bond between Cys localized in the M1-P1 linker [7, 13]. However, TASK subfamily members (TASK1, TASK3, and TASK5) do not have Cys in the M1-P1 linker. Instead, TASK1 utilizes hydrophobic interactions via a coiled-coil structure for dimerization [8]. In contrast, TALK2 possesses a Cys residue, which is expected to form a disulfide bound, but it does not contain the coiled-coil domain [8]. Therefore, these two mechanisms do not explain how TASK1 and TALK2 form a heterodimer. Actually, it has been shown that the disulfide bond is not required for the functional expression as homodimers of TWIK1 or TRAAK [8]. Furthermore, TRAAK does not make a heterodimer with TASK1 [9], despite both of these channels contain the coiled-coil domains [8]. THIK1 and THIK2 can form a heterodimer, even though these two channels have neither the Cys residue nor the coiled-coil domain [11]. These findings indicated that even one of these two mechanisms is not mandatory for some types of K_2P_ dimerization and that another unknown mechanism can be speculated for TASK1-TALK2 dimerization.

It has been reported that ^98^His in TASK1 and ^242^Lys in TALK2 are key residues responsible for sensing extracellular pH changes [29, 30]. In both cases, de-protonation releases pore blocking and enhances K^+^ flux. As shown in Fig 4, the TASK1-TALK2 heterodimer showed intermediate pH sensitivity between TASK1 and TALK2 homodimers, but with much gentler slope than homodimers. Interestingly, the data of TASK1-TALK2 were fitted by the sum of two Hill equations better than a simple Hill equation. Supposing the proportion of TASK1 (s) and TALK2 (1-s) current components as 0.4 and 0.6, respectively, the data were well described by the two-tier fitting curve. This imbalance in the proportion (0.4:0.6) may be due to the incomplete saturation of TALK2 activation by strong extracellular alkalinization even at pH 10.0 (See [31] and Fig 4). In contrast, it has been reported that TASK1-TASK3 heterodimer [9, 32] shows intermediate reactions between each homodimer, and the data were well fitted to a curve of single Hill equation. This contrast between TASK1-TALK2 and TASK1-TASK3 heterodimers is probably attributable to the difference between pH-sensitivity of each subunit forming the heterodimer. pK_1/2_ values of TASK1 and TASK3 are 7.5 and 6.8, i.e. ΔpK_1/2_ = 0.7 [32], and pK_1/2_ values of TASK1 and TALK2 are 7.7 and 9.6, and ΔpK_1/2_ = 1.9 (Fig 4). Thus, ^98^His in TASK1 and ^242^Lys in TALK2 may work independently within a heterodimer. It is notable that the combinational expression of TASK1-TALK2 heterodimer in addition to these two homodimers provides graded current activation in response to wider range of pH changes from pH 5.4 to pH 9.4. The physiological impact of the wider pH sensing, for example in pancreatic islet cells, remains to be clarified yet.

Sensitivity to halothane is well studied in TASK1, TASK3, and TREK1 [16, 24, 33]. Halothane binds to TASK1 through the ^243^VLRFMT^248^ domain just at the end of the T4 segment [24] and ^245^Arg is potentially significant [33]. Of note, this domain influences the binding of thyrotropin-releasing hormone [33], diacylglycerol [34], and A1899 (a TASK1 blocker) [35]. Using the TASK1-TASK3 tandem constructs, it has been clearly shown that “two” ^243^VLRFM/LT^248^ domains within a dimer are essential for effective potentiation by halothane but “one” alone shows decreased halothane-potentiated TASK currents [24, 33]. The pH-sensitive current in HEK293 cells expressing TALK2 was significantly reduced by halothane (Fig 5A). This phenomenon has been shown previously [14], while the precise mechanism is not yet known. As for TASK1-TALK2 heterodimers, 0.3 mM halothane increased TASK1-TALK2 currents (I/I_control_: 1.43) to the same extent as TASK1 currents (I/I_control_: 1.65). On the other hand, potentiating effects of 1.0 and 3.0 mM halothane on TASK1-TALK2 were smaller than those on TASK1 (Fig 5C), presumably due to the decrease in TALK2 current component by halothane. Therefore, it can be simply suggested that TASK1 and TALK2 subunits within dimers independently respond to halothane. Similar responses have been observed when isoflurane is applied to HEK293 cells expressing TASK1 (weakly inhibited), TASK3 (largely increased) or TASK1-TASK3 heterodimers (moderately increased) [32]. Thus, heterodimerization may provide unique characteristics distinctive from those of homodimers to K_2P_ channels and allow them to sense much larger variations of physiological stimuli.

Single molecular imaging suggested the protein expression of TASK1-TALK2 a heterodimer as well as homodimers in a human pancreatic cell line, QGP-1. It has been reported that TASK1 reduces cell excitability and hormonal release from β cells [4] and α cells [5]. Although these reports did not mention the roles of TALK2 in these cells, TALK2 may possibly be involved in hormonal secretion by modulating TASK1 activity through heterodimerization. Notably, the TASK1-TALK2 heterodimer exhibited distinctive pharmacological properties from those of the homodimers, as discussed above. Therefore, in intact cells, where TASK1 and TALK2 form heterodimers, specific modulators of TASK1 or TALK2 may exhibit different effects from those detected in cells heterologously expressing each homodimer. Thus, to develop novel drugs targeting K_2P_ channels, it will be potentially important to take heterodimers in target tissues into account.

## Supporting information

S1 Fig Original blots and gels in Fig 1A, 1B and 1C. (TIF)

S2 Fig Original blots in Fig 2E. (TIF)
