# Supplementary figures and images for: Heterodimerization of two pore domain K+ channel TASK1 and TALK2 in living heterologous expression systems
Source: PLoS One. 2017 Oct 10;12(10):e0186252. doi: 10.1371/journal.pone.0186252 (PMC5634629; doi:10.1371/journal.pone.0186252)

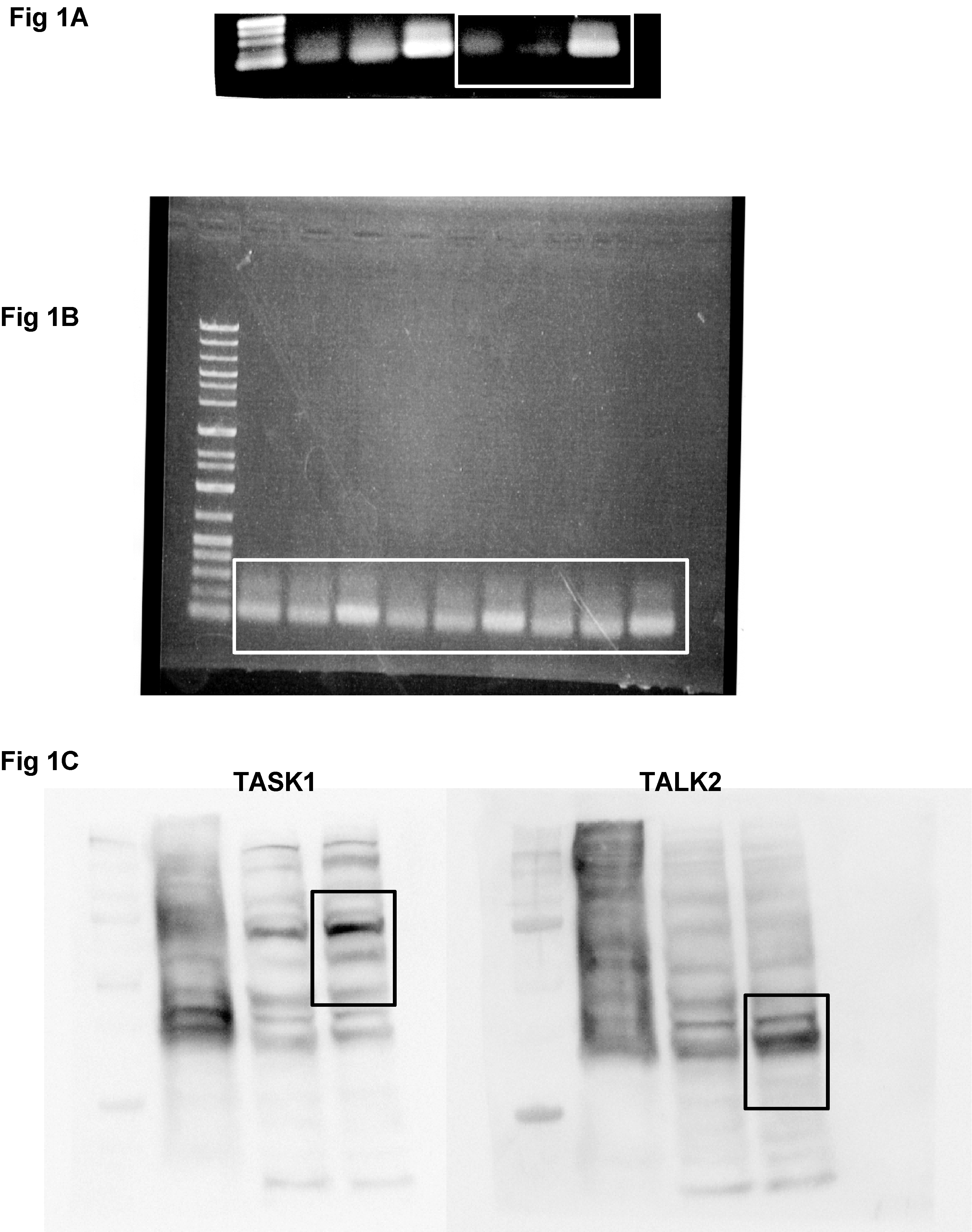

Supplement: S1 Fig — (TIF) [file pone.0186252.s001.tif]

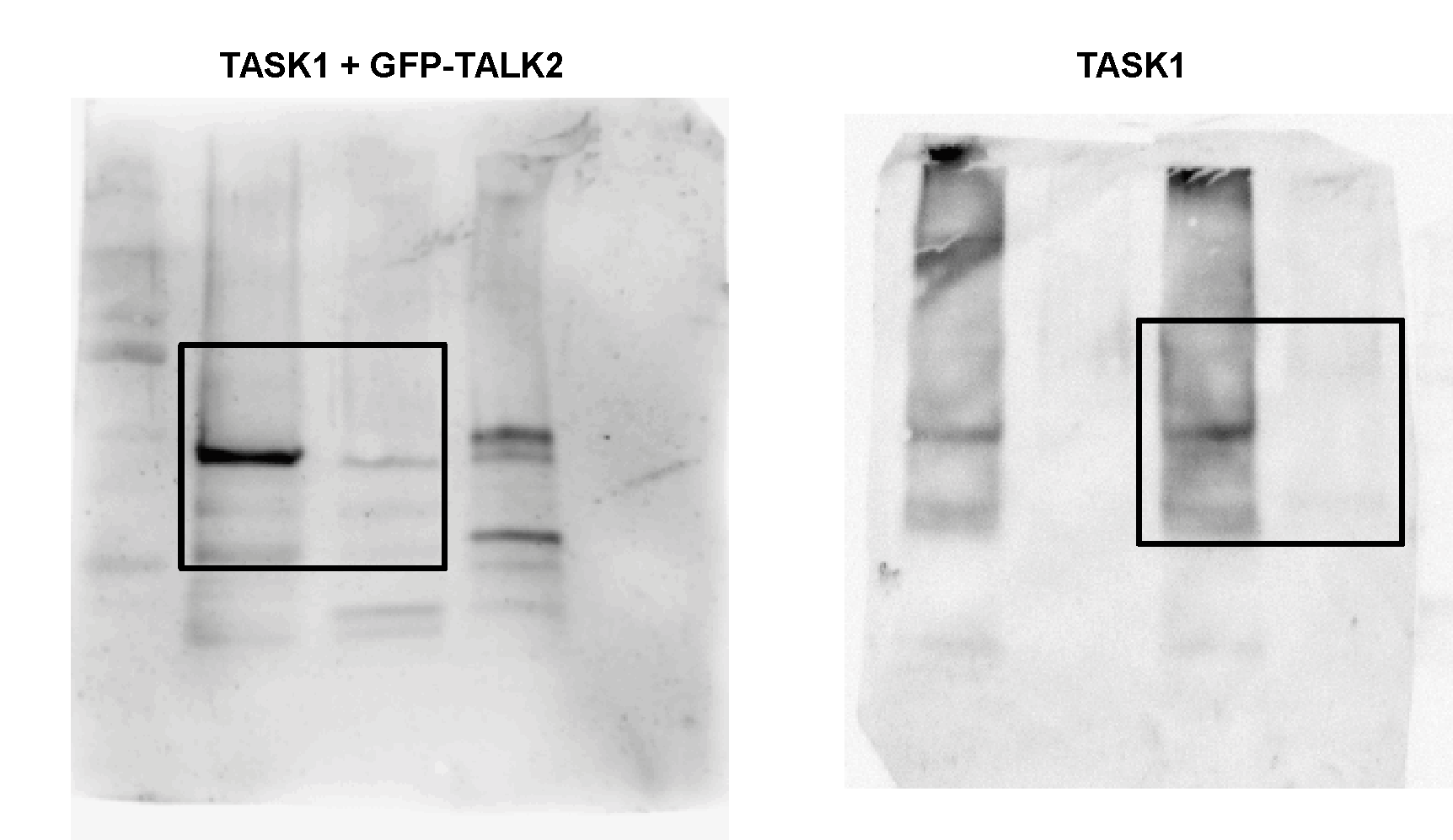

Supplement: S2 Fig — (TIF) [file pone.0186252.s002.tif]
